# Automated Image Analysis of Lung Branching Morphogenesis from Microscopic Images of Fetal Rat Explants

**DOI:** 10.1155/2014/820214

**Published:** 2014-08-28

**Authors:** Pedro L. Rodrigues, Nuno F. Rodrigues, Duarte Duque, Sara Granja, Jorge Correia-Pinto, João L. Vilaça

**Affiliations:** ^1^ICVS/3B's, PT Government Associate Laboratory, Guimarães, Braga, Portugal; ^2^DIGARC, Polytechnic Institute of Cávado and Ave, Barcelos, Portugal; ^3^Life and Health Sciences Research Institute (ICVS), School of Health Sciences, University of Minho, 4710-057 Braga, Portugal

## Abstract

*Background.* Regulating mechanisms of branching morphogenesis of fetal lung rat explants have been an essential tool for molecular research. This work presents a new methodology to accurately quantify the epithelial, outer contour, and peripheral airway buds of lung explants during cellular development from microscopic images. *Methods.* The outer contour was defined using an adaptive and multiscale threshold algorithm whose level was automatically calculated based on an entropy maximization criterion. The inner lung epithelium was defined by a clustering procedure that groups small image regions according to the minimum description length principle and local statistical properties. Finally, the number of peripheral buds was counted as the skeleton branched ends from a skeletonized image of the lung inner epithelia. *Results.* The time for lung branching morphometric analysis was reduced in 98% in contrast to the manual method. Best results were obtained in the first two days of cellular development, with lesser standard deviations. Nonsignificant differences were found between the automatic and manual results in all culture days. *Conclusions.* The proposed method introduces a series of advantages related to its intuitive use and accuracy, making the technique suitable to images with different lighting characteristics and allowing a reliable comparison between different researchers.

## 1. Introduction

Branching morphogenesis results on the creation of branched structures in the body and is a key and fundamental feature of several organs development and growth, such as lungs, pancreas, salivary glands, mammary glands, kidney, and prostate [1–3]. The lung branching morphogenesis (LBM) of fetal rat explants, grown in vitro, has been an essential tool in research of molecular and cellular development mechanisms. This methodology has been widely studied, at different gestational ages in vivo and in vitro, in many research centers due to its stability and versatility [4–7].

Usually, LBM analysis involves a morphometric analysis of lung explants differentiation and growth, during a 5-day period, using stereo microscope images acquired at 24-hour intervals. For each day, a comprehensive study of the branching pattern of embryonic lungs by quantifying the branches perimeter, area, outer contour, and number of peripheral airway buds is performed.

Although, over the past decade, there have been significant advances in understanding of the genetic control of lung development, to the best of our knowledge, all LBM studies are still performed by manual image quantification using generic 2D curves software. LBM analysis remains a time-consuming procedure, dependent on researcher expertise and error-prone. Often, it prevents the biological result comparison among different researchers, to deduce biological validations and theorems, due to ambiguous and inaccurate measurements [8]. Therefore, besides the different image processing approaches proposed in the biological research domain [9–18], none of these works are applied to LBM analysis preventing us from discussing the state-of-the-art technology to deal with the same problem.

Considering the literature pitfalls, we propose a new methodology capable of automatically performing the LBM morphometric analysis in order to reduce or even eliminate the researcher dependence, providing fast, robust, user-independent, and accurate results.

## 2. Methods

All methods were developed using C++, ITK (Segmentation & Registration Toolkit), and VTK (Visualization Toolkit). RGB images were acquired at the Life and Health Sciences Research Institute (ICVS) of School of Health Sciences, University of Minho, Portugal, using a stereo microscope (Olympus SZX16). Each CT slice has 768 × 576 pixels, with a pixel resolution of 40 *μ*m. All images were acquired at the ×20 magnification.

**Figure 1:**
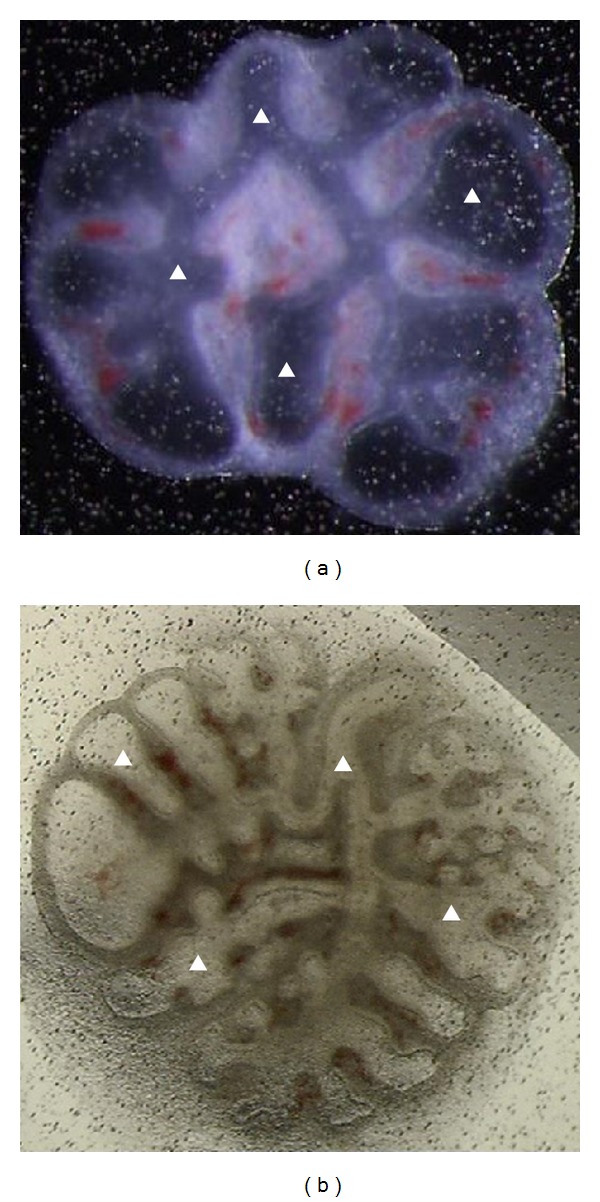
Images of lung explant grown in vitro with different stereo microscope lighting sources.

Depending on the biological laboratory trials, LBM images can be acquired with different color conditions (examples in Figures 1(a) and 1(b)). The computer application presented in this work was tested and validated for both kinds of images. The segmentation process assumes that the region of interest to be segmented is the union of one or more small primitive regions previously calculated from the input image. To this extent, different seeds were automatically calculated and placed within the lung epithelia allowing a multithreading cluster growth.

### 2.1. Preprocessing Filtering

The RGB input images were first converted to grayscale values by averaging and normalizing the 3-color components. Then, the grayscale image was input to an anisotropic diffusion algorithm [19] which reduces the noise spots corrupting the image.

**Figure 2:**
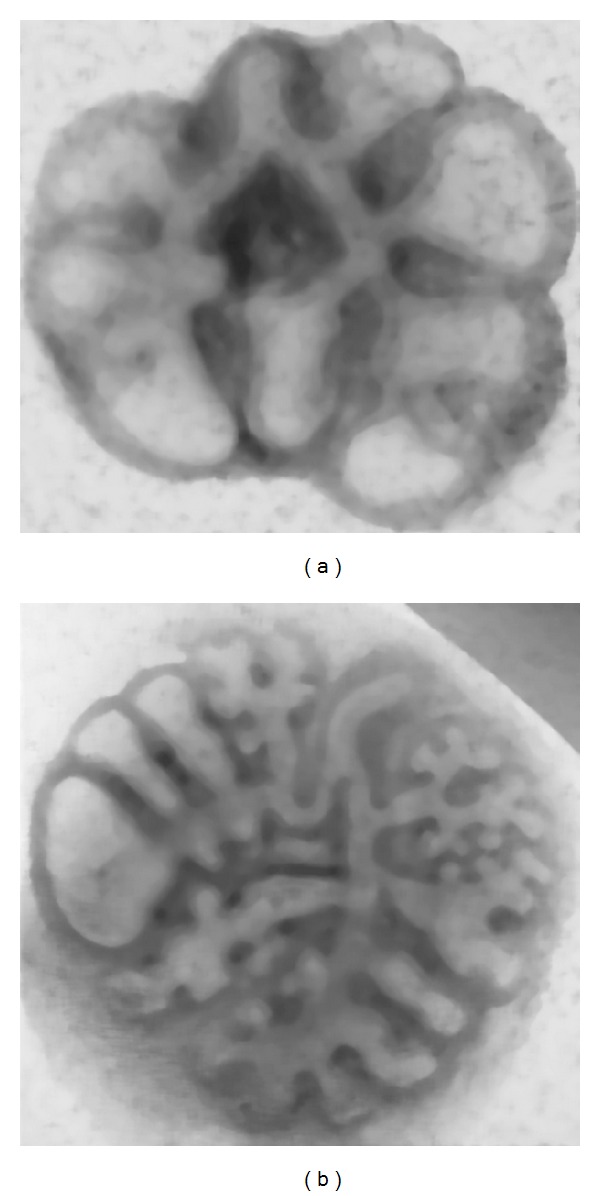
Anisotropic diffusion outcome for both images shown in Figures 1(a) and 1(b), respectively. The images were diffused according to Tukey's function using 80 iterations and an edge parameter of *σ* = 4.5 (determined experimentally in order to enhance the inner lung epithelia).

This algorithm depends on three parameters (empirically defined): the number of iterations, edge parameter (*σ*), and an edge-stopping diffusivity function *g*(*x*, *σ*), according to Tukey's function: *g*(*x*, *σ*) = (1/2)[1−(*X*/*σ*)^2^]^2^  if  |*x*| ≤ *σ* or *g*(*x*, *σ*) = 0, *f*|*x*| > *σ*. The anisotropic diffusion algorithm worked as a low-pass filter for noise reduction but preserving sharper boundaries and image contours, producing uniformity in the output image intensity (Figures 2(a) and 2(b)).

This outcome was used to automatically segment (1) the outer contour of the lung explant object and also (2) the inner epithelia by merging different clusters according to an image partitioning.

### 2.2. Lung Explant Outer Contour

An adaptive and multiscale thresholding technique was used to accomplish the segmentation of the whole lung explant object from the background.

Initially, a global threshold that maximizes the image entropy between a segmented object (the lung explant) and its background was automatically calculated. For that, consider an image *I* with *N* pixels, *I*(*i*) as the image intensity at position *i* (*i* = 1,2,…, *N*), and IMin and IMax equal to 0 and 255. Moreover, consider *T*_*I*_(*i*) as the result of a threshold algorithm with a threshold level at *I*(*i*), *E*_*O*_(*i*) as the entropy of the objects of *T*_*I*_(*i*), *E*_*B*_(*i*) as the entropy of the background of *T*_*I*_(*i*), and *E*_*T*_ as the total entropy (*E*_*B*_(*i*) + *E*_*O*_(*i*)). Ranging from IMin to IMax, the value of *T* was determined by the intensity *I*(*i*) that maximized *E*_*T*_.

Although this global threshold produces suitable contours in all lung objects with image properties as Figure 1(a), it fails for Figure 1(b) due to the irregularities, small contrast variability, and outer contour ambiguities.

**Figure 3:**
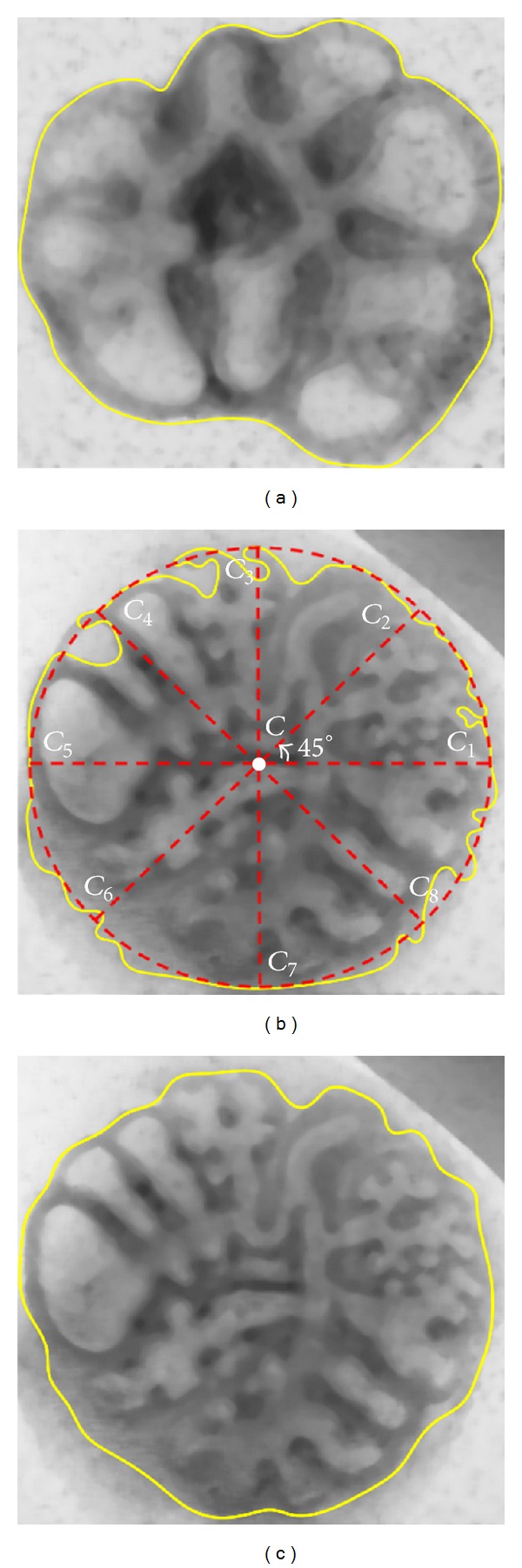
(a) Outer contour for Figure 1(a); (b) outer contour for Figure 1(b) and representation of the new strategy to redefine this initial contour; and (c) redefined counter for Figure 1(b).

Consequently, for this kind of images, the initial outer contour was redefined with the following steps:

- calculation of an initial binary object and its centroid (*C* in Figure 3(b)) is done;
- determination of different lines with origin at *C* (slopes incremented from 0° to 360° with 45° of step) is done;
- let *N* be the total number of lines (*N* = 8); calculate the distances (*D*_*i*_, *i* = 1,2,…, *N*) between the origin point and the one that intersects the initial contour (*C*_*i*_, *i* = 1,2,…, *N*);
- determine the distance average $\bar{D}=\sum_{i=1}^{i=N}D_{i}/N$; it defines circle with radius $\bar{D}/2$ and center at *C*;
- each point *C*_*i*_ corresponds to the center of a new circle with radius $\bar{D}/4$ that was used to automatically determine a local threshold level using the same entropy maximization criterion;
- the threshold value, for all pixels that were not inside of any circle with center at *C*_*i*_, was smoothly interpolated using a B-Spline approximation method described in [20];
- the resulting binary image allowed the determination of an iso-contour for the whole lung object; this contour was later smoothed with a Gaussian distribution producing a shrinking effect (Figure 3(c)).

### 2.3. Epithelial Segmentation

#### 2.3.1. Image Partitioning

**Figure 4:**
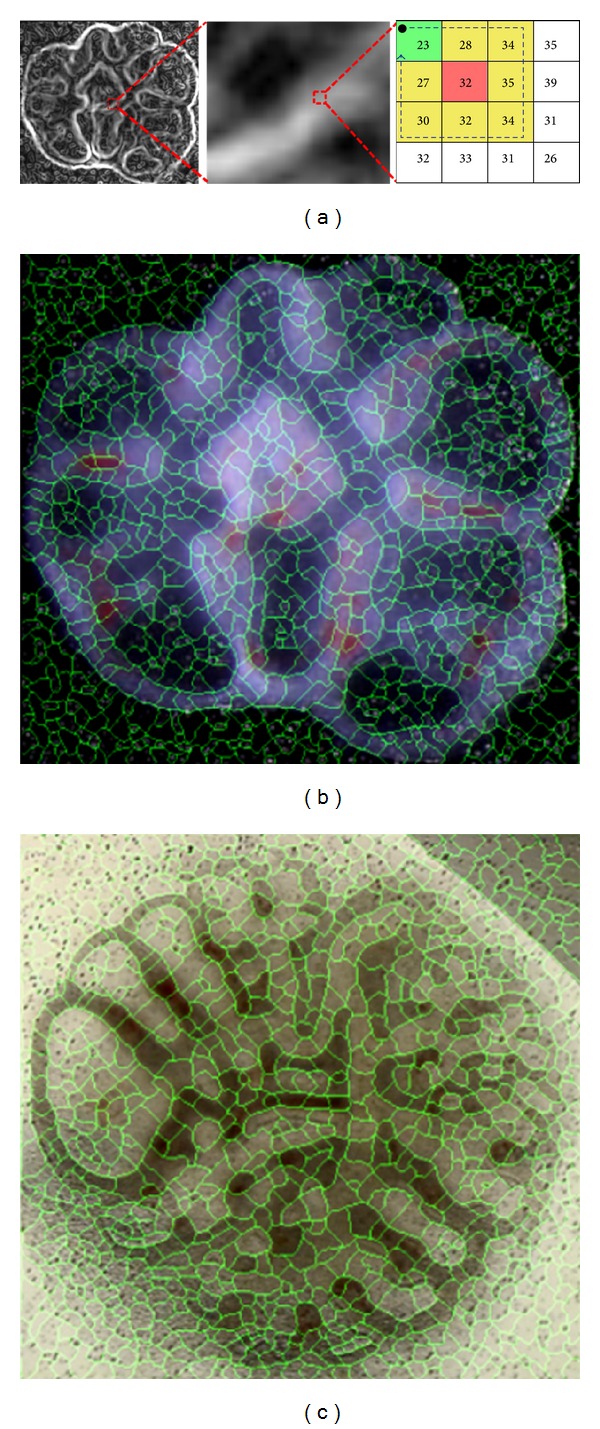
(a) Labeling process using the image gradient magnitude: (left) image gradient magnitude; (center) zooming image area; (right) the numbers are the pixel intensities, red is the stating pixel, green is minimum pixel intensity in the 8-connected neighborhood (in yellow), and the arrow is the searching direction. (b) and (c) are image partitioning in rat lung explants for Figures 1(a) and 1(b).

The image gradient magnitude of the filtered image was input to an algorithm that divides the input image into small regions.  Figure 4(a) shows how the different regions were labelled by a starting point (red, Figure 4(a)) and follow the flow line, whose direction was the gradient of minimum local intensity. The minimum gradient (green, Figure 4(a)) path of each pixel *p* of the input image was tracked by recursively selecting a pixel *q* in the 8-connected neighborhood (yellow, Figure 4(a)). If more than one pixel *q* exist, the last pixel found was taken, considering *p* as a reference pixel. Every pixel *q* along the path is marked as a local minimum of the gradient magnitude and assigned a distinct label.

In the end, the whole image was divided into primitive regions. Each region shares the same statistical properties and the boundaries coincide with the ridges of the gradient magnitude surface (Figures 4(a) and 4(b)).

#### 2.3.2. Clustering Regions

*Creating Seeds*. The image partitioning contains a set of nonoverlapping regions. Although the probability of having region boundaries corresponding to boundaries of important objects increases with oversegmentation, it can also create many insignificant boundaries. This stage describes how one dealt with this problem and the inner lung epithelium was automatically determined. Briefly, the procedure consists of the identification and clustering of similar primitive regions.

**Figure 5:**
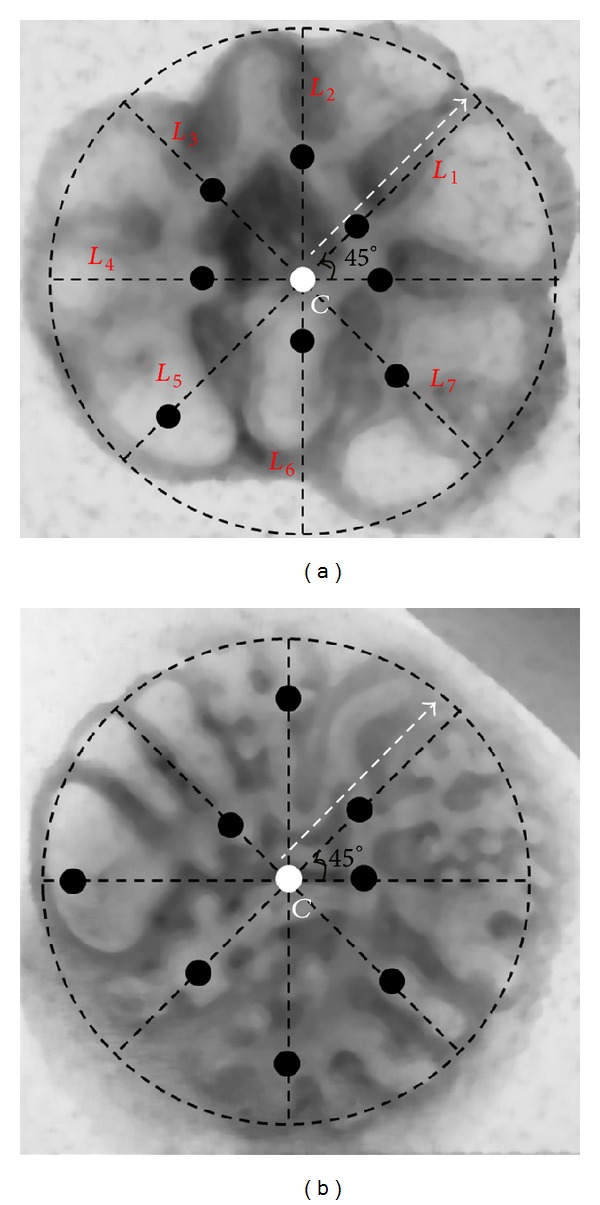
Overview of the clustering procedure. The white circle represents a kernel at center *C*; the black circles represent the initial seeds for clustering growth.

Each cluster starts growing from different seeds, initialized within the lung epithelia, along different lines *L*_*i*_ (with *i* = 1,2,…, 8 (total number of lines), Figure 5(a)).

First, a neighborhood *N*_*i*_ (with *i* = 1,2,…, 8, white circle in Figure 5) defines a kernel with center at centroid *C*, circle shape, and radius of 8 pixels. An iterative process transverses each line *L*_*i*_ with *N*_*i*_ (white arrow in Figure 5), while it calculates the average distribution of the kernel neighborhood originating different candidate seeds. The final seed for clustering grown will be the one where the average distribution within the kernel was minimum (black circles in Figure 5 defining a seed *S*_*i*_).

The regions belonging to each seed *S*_*i*_ were used to calculate initial statistical properties of the epithelia (centroid, average distribution, minimum and maximum values, region edges, region neighbors, and entropy) that were used for clustering growth.

*Clustering Growth*. The merging procedure was based on the similarity between regions formulated mathematically as a local optimization problem using the minimum description length principle [21]. If any primitive region is neighbor of a cluster, initialized at a seed *S*_*i*_, a decision rule will state if it should be included or not.

**Figure 6:**
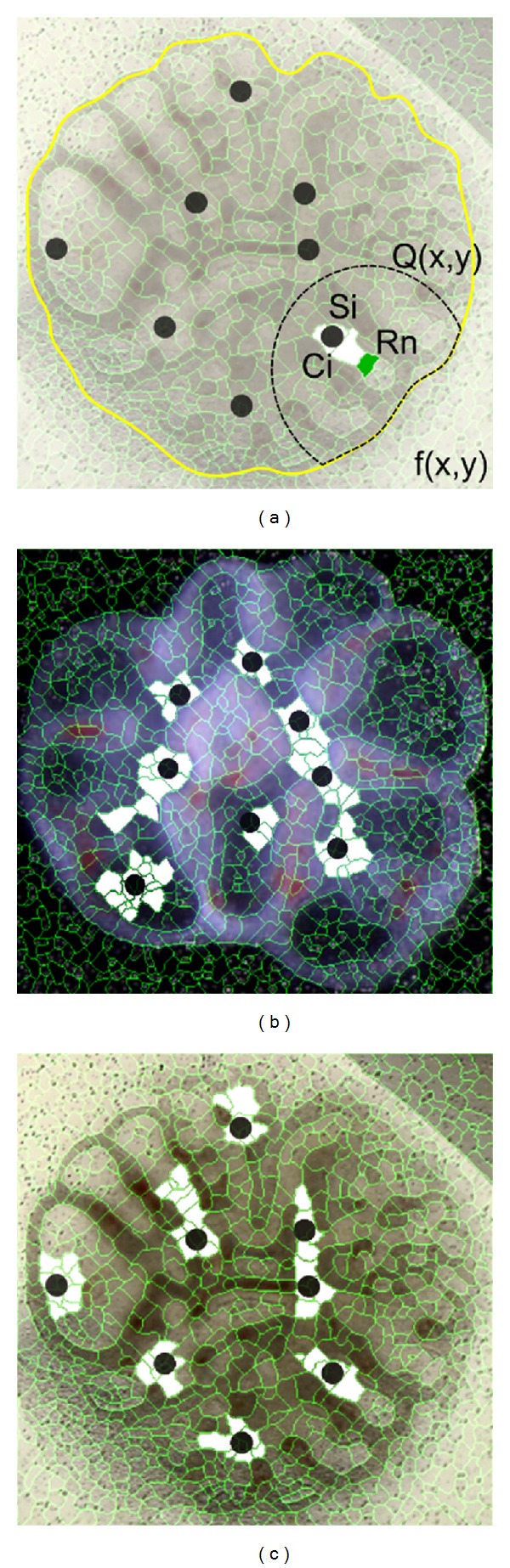
Overview of the clustering growth procedure: (a) shows how a local region *Q*(*x*, *y*) is calculated in a cluster *C*_*i*_ with seed *S*_*i*_ with center at *R*_*n*_ (query region tested whether its inclusion is suitable using the minimum description length principle); (b) and (c) show a multicluster growth simultaneously.

Let *f*(*x*, *y*) be a two-dimensional function that denotes the 2D input image with *k* constant regions and *I*_*i*_  (*i* = 1,2,…, *k*) the original image intensity in the *i*th region. Let *Q*(*x*, *y*) be a region of *f*(*x*, *y*) with circular shape that includes a maximum number of regions (*n* = 100, experimentally calculated), located within the outer contour and center at the new query region *R*_*n*_ (tested whether its inclusion in the cluster *C*_*i*_ is adequate) (Figure 6(a)). Using a region *Q*(*x*, *y*) with a limited number of primitive regions reduces the probability to merge statistically outlier regions, providing a truer picture of the inner epithelia.

According to the minimum description length, the image data was coded in order to determine the total number of bits necessary to encode the region *Q*(*x*, *y*) given by *B*_*R*_ = ∑_*i*_ *B*_*I*_(*R*_*i*_) + *B*_*B*_(*R*), where

1. *B*_*I*_(*R*_*i*_) is the total number of bits needed to describe the image intensity for each region given: *B*_*I*_(*R*_*i*_) = *nR*_*i*_ *H*(*R*_*i*_) (with *nR*_*i*_ being the number of pixels and image intensity entropy within *R*_*i*_);
2. *B*_*B*_(*R*) is the number of bits needed to code the region boundary information given by *B*_*B*_(*R*) = *N*_*r*_(*R*) · *b*_1_ + *N*_*b*_(*R*) · *b*_2_ (with *N*_*r*_(*R*) being the number of regions in *R*, *N*_*b*_(*R*) the total boundary length of the partitioning, *b*_1_ the number of bits required to code the starting point, and *b*_2_ the number of bits required to code each element of the boundary chain code).

As a local optimization problem, the inclusion of new region *R*_*n*_ in the cluster *C*_*i*_ aims at providing the largest positive description length gain *G* in *Q*(*x*, *y*) at each step with local optimization.

If a new region is merged in the cluster, the cluster will have more pixels. Hence, more bits are needed to encode the cluster image intensities. However, the common boundary segment between *R*_*n*_ and *C*_*i*_ disappears and the total description length might decrease, since the number of bits needed to encode the new region boundary information decreases.

With *nb*(*R*_*n*_, *C*_*i*_) being the number of common boundary elements of *R*_*n*_ and *C*_*i*_ and *C*_new_ the new cluster resulting from the merging procedure, the value of description length gain *G* associated with this merging is given by

(1) G= nRnH(Rn)+nCiH(Ci)−nCnew H(Cnew)+b1+nb(Rn,Ci)·b2.

If *G* > 0, the image intensities between *R*_*n*_ and *C*_*i*_ are quite similar, whereby these regions belong to the same object. Thus the entropy increase is compensated by the elimination of the common boundary, and then these two regions should be merged in order to minimize *B*_*B*_(*R*).

### 2.4. Buds Counting

The number of peripheral buds was counted based on the lung inner epithelial skeletonizing [22]. The skeletonizing is performed by removing the boundary and corner points of the epithelial object, until only the skeleton remains as white pixels.

**Figure 7:**
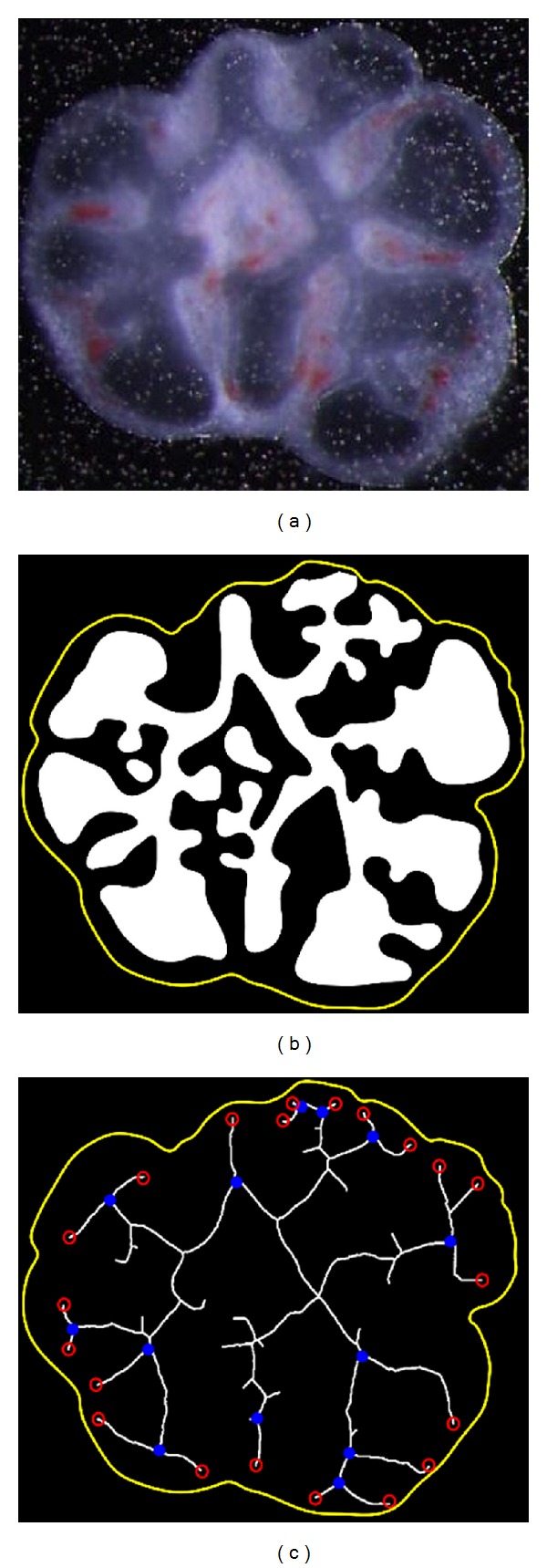
(a) Input image; (b) outer contour and segmented inner lung epithelia in white; and (c) skeleton of the inner epithelia; branched ends (circles in red); branched ends parents (circles in blue).

The number of peripheral buds was determined as the number of parents (circles in blue, Figure 7(c)) of the skeleton branched ends (circles in red, Figure 7(c)). Only the branched ends located near the outer contour (distancing less than 25% of the axis that transverses the lung object vertically) were considered.

## 3. Results

The performance of this new computer algorithm was tested and validated in a total of 210 images: 90 (18 images for each day of culture) with image conditions as Figure 1(a) and 120 (24 images for each day of culture) with image conditions as Figure 1(b). All images were previously segmented by three experienced researchers: each user manually segmented the inner epithelia and outer contour and counted the number of peripheral buds.

The performance of the manual quantification was accessed by comparing the results among different researchers. The inner epithelia and outer counter were evaluated using the dice similarity coefficient (DSC) that quantifies the spatial overlap between different segmentations as follows:

(2) $$\begin{array}{c} \text{DSC}\left(A,B\right)=\frac{2\left|A\cap B\right|}{\left|A\right|+\left|B\right|}=\frac{2\text{TP}}{2\text{TP}+\text{FP}+\text{FN}}, \end{array}$$

where TP is the number of true positives, FP the false positives, and FN the false negatives. The DSC score ranges from 0%, indicating no spatial overlap between sets of binary segmentation results, to 100%, indicating complete overlap.  Table 1 shows the mean DSC score and error difference for the LBM morphometric analysis among three researchers.  Table 2 shows the mean differences when the automatic method was compared to each manual result. The* peripheral buds error* is the difference average error between users in Table 1 and difference average error between manual and automatic methods in Table 2.

Best results were obtained in the first two days for both types of images with less standard deviations. High similarities between the manual and automatic procedure were not easily obtained for the last two days of culture, due to the increased number of peripheral airway buds and lung architecture complexity.

Statistical analysis using ANOVA test (under SPSS Windows version 17.0 software where *P* values lower than 0.05 were accepted as significant) shows nonsignificant differences between the automatic and manual results (inner epithelia and outer counter) in all culture days. On the other hand, the error of peripheral buds was nonsignificant only on the first three days of culture. Nonsignificant differences were found between researchers. The time for LBM morphometric analysis was reduced to 1-2 seconds/image in contrast to the manual one (2-3 minutes/image).

**Figure 8:**
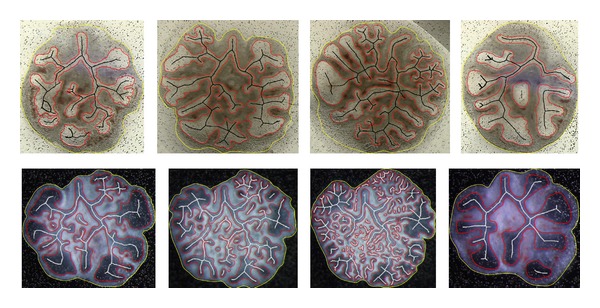
LBM morphometric analysis for different type of images: outer counter in yellow, inner epithelia in red, and branched skeleton in white.

Results from different images regarding images with different lighting conditions are shown in Figure 8.

A user interface was also developed that allows the user to spatially understand the microscope image and rapidly produce an automatic segmentation. If necessary, manual editing may be used to correct automatic segmentation results. Although this manual editing (used to eliminate false merges) improves DSC scores (>98%) and decreases the standard deviations, it also adds user dependence and slows the morphometric analysis (depending on the editing degree).

This strategy has been used at the ICVS research lab to study and analyze the effect of different concentrations of a specific inhibitor in lung branching morphogenesis.

## 4. Discussion

A computer application was developed, providing an automatic procedure to enable a fast LBM analysis. The morphometric analysis efficiency and robustness were increased while time consumption, user dependence, and subjectivity were decreased or even eliminated. The observer variability was eliminated since all regions were computed and merged automatically.

The segmentation rate depends on the number of regions needed to be merged to select the entire lung epithelial region. However, the processing time was always about 98% less than the manual one.

The merging procedure was essential to achieve a good segmentation, since a significant amount of regions were initially created by the partitioning algorithm. The automatic seed selection was suitable to segment the inner epithelia using the minimum description length criterion that selectively cluster regions based on their image intensity distribution similarity.

It was seldom observed that clusters merged dissimilar regions due to ambiguity and lack of definition of the inner lung explants contours. The worst results were obtained in the last two days of culture (Table 2), whose images present low details and perceptibility, and the branched ramifications increase drastically. However, the usage of a local image region, centered at the query region, tested whether its inclusion is suitable using the minimum description length principle. This allowed a reliable cluster to grow along epithelial object that changes gradually over space.

The local threshold was efficient to automatically delineate the outer contour with high DSC scores in images with lighting characteristics presented in Figure 1(b).

The main difficulties found in the segmentation procedure were the contrast ambiguity and variances of inner epithelia, complexity of the branched shape, and size, and the presence of neighborhood regions with the same density. Moreover, response to drugs and biological markers and different culture medium can induce image intensity variations and shadows near the outer contour.

These difficulties were more evident at the last two days of culture where DSCs are lower, even when comparing the results from different researchers. However, the inner epithelial segmentation was always segmented with scores around 90%, even at the last two culture days, indicating successful segmentation.

However one has to recognize some limitations within this work. The proposed algorithm overpredicts the number of peripheral buds with statistical significance with manual counting. However, peripheral buds counting is a controversial issue, without any stated method. As presented in Table 1, the coefficient of variation (defined as the ratio between the standard deviation and mean) among researchers is around 1, showing that there is no coherency between them. With this methodology, one aimed to broaden and generalize this procedure among different researchers. However, further biological studies are required to evaluate its suitability and reliability for lung branching analyses.

## 5. Conclusions

This work presents an automatic segmentation procedure and implementation, providing a technique for LBM morphometric analysis. The proposed method introduces a series of advantages related to its intuitive use and accuracy, making the technique suitable to images with different lighting characteristics. The total number of human decisions, time consumption, and user dependence were significantly decreased.

Due to its automatization nature, this application allows a reliable comparison of different researchers' results and the possibility for more than one researcher to perform the same LBM study.

Results show that further work is needed regarding the merging procedure and the development of image enhancement techniques to improve inner lung epithelial contrast, mainly in the last days of culture. Moreover, a new strategy must be developed for counting the number of peripheral airway buds. Finally, this method has the potential to be used in different research environments to improve research outcomes.

## Figures and Tables

**Table 1:** DSC scores of LBM analysis comparing different researchers results when the study was made in different culture days and image of types.

	Days of culture					
		1	2	3	4	5
Inner epithelial DSC %	A images	91.77 ± 5.13	88.99 ± 2.03	87.93 ± 5.44	87.97 ± 6.76	87.60 ± 7.94
	B images	86.91 ± 5.83	86.45 ± 4.23	87.26 ± 4.72	85.53 ± 8.23	85.78 ± 9.69

Outer counter DSC %	A images	97.87 ± 4.65	97.32 ± 5.84	97.73 ± 4.89	96.19 ± 7.00	97.15 ± 5.46
	B images	94.24 ± 2.90	94.97 ± 5.89	93.97 ± 7.42	91.92 ± 9.25	90.82 ± 10.15

Peripheral buds error	A images	0.5 ± 0.2	1.2 ± 0.7	2.5 ± 0.8	2.5 ± 1.2	3.5 ± 3.2
	B images	0.4 ± 0.6	1.8 ± 1.3	2.3 ± 1.2	2.4 ± 2.1	4.2 ± 5.6

**Table 2:** DSC scores of LBM analysis comparing the manual and automatic methods when the study was made in different culture days and image of types.

	Days of culture					
		1	2	3	4	5
Inner epithelial DSC %	A images	92.85 ± 3.87	91.54 ± 6.12	90.87 ± 6.88	88.80 ± 9.96	86.72 ± 8.75
	B images	89.99 ± 5.32	87.74 ± 9.79	87.06 ± 8.29	82.26 ± 14.64	79.15 ± 13.33

Outer counter DSC %	A images	98.09 ± 2.48	99.05 ± 3.49	97.56 ± 5.31	96.28 ± 3.75	97.12 ± 2.03
	B images	95.48 ± 6.61	94.54 ± 8.37	95.89 ± 12.30	94.64 ± 13.46	94.71 ± 13.42

Peripheral buds error	A images	1.0 ± 1.8	2.6 ± 2.3	4.7 ± 5.8	4.4 ± 6.0	10.3 ± 7.7
	B images	1.3 ± 2.4	2.2 ± 3.1	6.5 ± 5.3	7.8 ± 6.2	12.5 ± 7.4
